# Early treatment with IgM-enriched intravenous immunoglobulin does not mitigate critical illness polyneuropathy and/or myopathy in patients with multiple organ failure and SIRS/sepsis: a prospective, randomized, placebo-controlled, double-blinded trial

**DOI:** 10.1186/cc13028

**Published:** 2013-10-02

**Authors:** Richard Brunner, Walter Rinner, Christine Haberler, Reinhard Kitzberger, Thomas Sycha, Harald Herkner, Joanna Warszawska, Christian Madl, Ulrike Holzinger

**Affiliations:** 1 Department of Medicine III - Division of Gastroenterology and Hepatology, Medical University of Vienna, Waehringer Guertel 18-20, 1090 Vienna, Austria; 2 Department of Neurology, Medical University of Vienna, Waehringer Guertel 18-20, 1090 Vienna, Austria; 3 Institute of Neurology, Medical University of Vienna, Waehringer Guertel 18-20, 1090 Vienna, Austria; 4 Department of Emergency Medicine, Medical University of Vienna, Waehringer Guertel 18-20, 1090 Vienna, Austria

## Abstract

**Introduction:**

Critical illness polyneuropathy and/or myopathy (CIPNM) is a severe complication of critical illness. Retrospective data suggest that early application of IgM-enriched intravenous immunoglobulin (IVIG) may prevent or mitigate CIPNM. Therefore, the primary objective was to assess the effect of early IgM-enriched IVIG versus placebo to mitigate CIPNM in a prospective setting.

**Methods:**

In this prospective, randomized, double-blinded and placebo-controlled trial, 38 critically ill patients with multiple organ failure (MOF), systemic inflammatory response syndrome (SIRS)/sepsis, and early clinical signs of CIPNM were included. Patients were randomly assigned to be treated either with IgM-enriched IVIG or placebo over a period of three days. CIPNM was measured by the CIPNM severity sum score based on electrophysiological stimulation of the median, ulnar, and tibial nerves on days 0, 4, 7, 14 and on the histological evaluation of muscle biopsies on days 0 and 14 and ranged from 0 (no CIPNM) to 8 (very severe CIPNM).

**Results:**

A total of 38 critically ill patients were included and randomized to receive either IgM-enriched IVIG (n = 19) or placebo (n = 19). Baseline characteristics were similar between the two groups. CIPNM could not be improved by IVIG treatment, represented by similar CIPNM severity sum scores on day 14 (IVIG vs. placebo: 4.8 ± 2.0 vs. 4.5 ± 1.8; *P* = 0.70). CIPNM severity sum score significantly increased from baseline to day 14 (3.5 ± 1.6 vs. 4.6 ± 1.9; *P* = 0.002). After an interim analysis the study was terminated early due to futility in reaching the primary endpoint.

**Conclusions:**

Early treatment with IVIG did not mitigate CIPNM in critically ill patients with MOF and SIRS/sepsis.

**Trial registration:**

Clinicaltrials.gov: NCT01867645

## Introduction

Critical illness polyneuropathy (CIP) and critical illness myopathy (CIM) are serious complications of severely ill patients [1].

CIP is an acute and primarily distal axonal sensory-motor polyneuropathy affecting mainly lower extremities and respiratory muscles [2]. As in some patients when primarily the muscles are affected, the term critical illness myopathy (CIM) was established [1]. However, the differentiation between CIP and CIM is difficult. Therefore, and due to the frequent association of both, the term critical illness polyneuropathy and/or myopathy (CIPNM) was introduced in 2000 [3]. Moreover, electrophysiological and histological findings of CIP and CIM disclose a significant overlap of these two entities [4].

In prospective studies, about 60 to 80% of patients with multiple organ failure (MOF) with or without sepsis or systemic inflammatory response syndrome (SIRS) presented with CIPNM [5-7]. In patients with septic shock [8] or severe sepsis and coma [9] the prevalence may reach up to 100%. In the majority of patients with sepsis a combination of both CIP and CIM was described [10].

Independent risk factors for CIPNM are, amongst others, severity of illness, duration of MOF with or without SIRS, duration of vasopressor and catecholamine support, hyperglycemia and duration of intensive care unit (ICU) stay [1].

The clinical features of CIP and CIM are almost identical and include muscle weakness and atrophy primarily of the lower limbs and respiratory muscles, delayed weaning from the respirator not explained by pulmonary or cardiovascular findings, and prolongation of the mobilization phase [1]. Moreover, a number of complications, such as pneumonia, deep vein thrombosis and pulmonary embolism may be attributed - at least in part - to CIPNM [11]. On neurological examination, decreased or absent tendon reflexes, especially with CIP, muscular atrophies and symmetrical flaccid tetraparesis are present [1].

The gold standards used to diagnose CIPNM are electrophysiological stimulation (EPS) and muscle biopsy. Characteristically, electromyography (EMG) and nerve conduction velocity (NCV) studies demonstrate the preservation of the speed of impulse in the presence of decreased compound muscle (CMAP) and sensory nerve (SNAP) action potential amplitudes [12]. These findings are highly consistent with a relatively pure axonal polyneuropathy. Furthermore, EMG discloses signs of denervation like fibrillation potentials and positive sharp waves in a widespread distribution. For the definite diagnosis of CIM and to differentiate between CIP and CIM the histological assessment of a muscle biopsy is the preferable method [1].

For CIPNM no specific pathogenic-based therapy is proven. For prevention, sepsis should be treated with maximum effort, including intensive insulin therapy (IIT) [13]. Muscle relaxants and corticosteroids should be administered at the lowest doses needed, whereas the potentially detrimental effect of the latter has been controversially discussed [14].

However, there is weak evidence from a retrospective chart analysis of prospectively collected data, that early IgM-enriched IVIG application may prevent CIPNM [15].

IVIG contains natural polyreactive antibodies derived from human plasma of healthy donors directed against endogenous and exogenous antibodies, immunomodulating peptides and various cytokines [16].

The pathophysiologic rationale for using IVIG to treat CIPNM is based on the association of CIPNM with pro-inflammatory cytokines accompanied by increased E-selection expression [3,17]. This favors the accumulation of neurotoxic factors in the endoneurium and causes extravasation of activated leukocytes both resulting in neuron damage [18]. Furthermore, elevated cytokine levels directly induce muscle protein damage via activation of calpain and ubiquitine-proteasome [14]. The anti-inflammatory and immunomodulating properties of IVIG may attenuate the local immune activation on both the cellular and the humoral level [16].

Therefore, we aimed to investigate the use of IVIG in the early treatment of CIPNM in critically ill patients in a prospective, randomized, double-blinded and placebo-controlled setting.

## Material and methods

### Trial design and setting

This prospective, randomized, double-blinded, placebo-controlled trial was conducted in an eight-bed medical ICU at the University Hospital of Vienna, Austria.

### Participants

Critically ill patients with MOF (failure of two or more organs), a SIRS/sepsis diagnosis, and first clinical evidence for CIPNM were randomized. Organ failure was defined as a cardiovascular system dysfunction (systolic blood pressure <90 mmHg or mean arterial pressure <70 mmHg), kidney dysfunction (urine output <0.5 ml/kg body weight/hour for one hour, despite adequate fluid resuscitation), respiratory system dysfunction (ratio of PaO2 to FiO_2_ <250 in the presence of other dysfunctional organs or systems), hematologic dysfunction (platelet count <80.000/mm^3^ or decreased by 50% in the three days preceding enrollment in the absence of liver cirrhosis or previously known hematological disease), or metabolic dysfunction (unexplained metabolic acidosis: pH <7.30 or base deficit >5.0 mmol/L in association with a plasma lactate level >1.5 times of the upper normal limit).

Clinical signs of CIPNM were defined as decreased tendon reflexes, signs of incipient muscular atrophy, decreased muscle strengths in responsive and co-operative patients, or facial grimacing but reduced or absent movement of limbs after induction of a painful stimulus by nail bed compression as assessed by a clinical neurologist. The diagnosis of “clinical signs of CIPNM” was met if one or more of these features were found. The examinations were quantified in absolute measures, as well as compared to previous examinations of the same patient, if applicable.

Exclusion criteria were age <18 or >80 years, body weight >135 kg (due to potentially impaired quality of the electrophysiology examination), pregnancy or breast-feeding, known absolute IgA-deficiency(*), known IVIG-intolerability(*), pre-existing neuromuscular disorders(*) (ICD-10: G70 to G73), pre-existing severe polyneuropathy(*) (ICD-10: G61 to G63), known diseases of the peripheral nervous system(*) (ICD-10: G60 and G64), pre-existing disease of the central nervous system with relevant impairment of the motor function(*) (ICD-10: G10 to G13, G20 to G26, G35 to G37, G80 to G83), relevant pulmonary edema secondary to severe heart failure, survival expectancy <28 days based on an uncorrectable medical condition, moribund state with imminent death, HIV infection in association with a last known CD4^+^ count of <50/mm^3^(*), and requirement of chronic ventilator support for non-respiratory reasons (*). These exclusion criteria were applied to information from the medical history of the patient.

### Interventions

**Figure 1:**
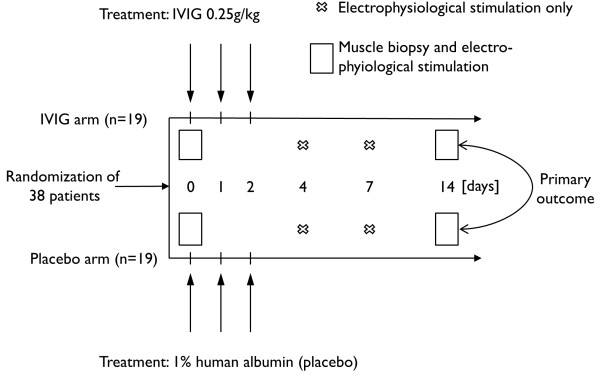
**Study timeline.** Patients with multiple (≥2) organ failure and a diagnosis of SIRS/sepsis were randomized to be treated either with intravenous immunoglobulins (IVIG) or human albumin (placebo) for three consecutive days. Critical illness polyneuropathy and/or myopathy (CIPNM) was assessed at baseline (Day 0) and on Day 14 by electrophysiological stimulation and histological assessment of a muscle biopsy. Primary endpoint was a CIPNM severity sum score on Day 14.

Randomized patients were treated either with IgM-enriched IVIG (Pentaglobin®, Biotest Pharma GmbH, Dreieich, Germany) or with human albumin 1% (Biotest Pharma GmbH, Dreieich, Germany) as placebo at a dose of 0.25 g/kg body weight/day as a continuous intravenous infusion at a rate of 2 g/h over a period of three days (Figure 1). Treatment was started immediately after all patient selection criteria, including clinical signs of CIPNM, were met.

### Outcomes

The primary outcome was to assess the effect of early IVIG versus placebo to mitigate CIPNM in critically ill patients as assessed by the CIPNM severity sum score on Day 14. CIPNM severity sum score is a combined endpoint consisting of the CIP and the CIM scores determined on Day 0 (baseline) and after treatment (Day 14).

CIP was determined by EPS of the median, ulnar and tibial nerves on days 0, 4, 7 and 14 using a Nicolet Viking IV (Nicolet Biomedical, Fenton, MO, USA) apparatus. CIP was graded based on the CMAP amplitude size according to the following scheme. CMAP amplitude ≥4,000 μV was considered as normal (score = 0), CMAP amplitude ≥3,000 μV and <4,000 μV as mild CIP (score = 1), CMAP amplitude ≥2,000 μV and <3,000 μV as moderate CIP (score = 2), CMAP amplitude ≥1,000 μV and <2,000 μV as severe CIP (score = 3), and CMAP amplitude <1,000 μV as very severe CIP (score = 4). For each day the nerve with the highest CIP score value was used for further calculations.

CIM was semi-quantitatively scored by an independent blinded neuropathologist according to the histological and ultrastructural findings of the skeletal muscle biopsy specimens taken on days 0 and 14.

The percutaneous biopsies in all patients were taken from the *Musculus vastus lateralis* by the same clinician according to a standardized protocol. In case of a second biopsy (on Day 14), the biopsy was performed on the contralateral muscle. The biopsy site was on the straight line between the great trochanter and the lateral condyle of the femur exactly 20 cm proximal of the lateral condyle. First, a small sterile field was prepared and local anesthetic was applied into the (sub)cutaneous area up to the *Fascia lata*. After the incision of the cutis at a 90° angle the area up to the *Fascia lata* was dissected out under visual control. Second, the muscle was biopsied using a Bergstroem muscle biopsy needle (Stille, Stockholm, Sweden).

Muscle tissue was snap frozen and a small part fixed in glutaraledhyde and embedded in resin. The panel of stainings, including HE, ATPase (pH 4.3), Oil-Red O, PAS, Gomori Trichrome, NADH and combined COX-SDH, was performed on the cross-cut frozen sections. Furthermore, sections were stained with antibodies against myosin slow and fast (Novocastra, Milton Keynes, UK, 1:100), γ sarcoglykan (Novocastra, 1:200), and N-terminal utrophin (Novocastra, 1:200). Thick sections of 4 μm were cut of the resin embedded tissue and stained with toluidine blue.

The quantification of myopathy was based on characteristic features of acute myopathy in intensive care, namely type II fibre atrophy (numerous scattered angular, atrophic fibers identified as type 2 fibers by ATPase and myosin stains), muscle necrosis, and selective loss of myosin filaments and scored as follows [4,14,19]: no signs of myopathy (score = 0), signs of mild myopathy (score = 1), signs of moderate myopathy (score = 2), signs of severe myopathy (score = 3), and signs of very severe myopathy (score = 4).

Hence, the CIPNM severity sum score consisting of the CIP and CIM scores determined on days 0 and 14 ranged from 0 (no CIM, no CIP) to 8 (very severe CIP, very severe CIM).

Secondary outcomes were to assess the effect of early IVIG versus placebo on mortality from any cause within a 28-day period and length of the ICU stay. Furthermore, we investigated the course of CIPNM from baseline to Day 14 in all patients.

### Sample size

The software PASS 11 (NCSS, Kaysville, UT, USA) was used for sample size calculation. Group sample sizes of 2 × 30 patients achieve 81% power to detect a difference in CIPNM sum score of 1.5 between the intervention group (estimated score of 4.0) and the control group (estimated score of 2.5) given standard deviations of 2.0 and at a two-sided significance level (alpha) of 0.05 using a Mann-Whitney test assuming that the actual distributions are equal.

### Randomization

The software “Randlist” (University of Gottingen, Germany) was used for randomization. Patients were stratified by Acute Physiology and Chronic Health Evaluation III (APACHE III) scores (low risk: ≤60; high risk: >60) and random permuted blocks within strata were generated (block size = 6). A person not otherwise involved in this study randomized patients 1:1.

IVIG and human albumin were supplied in a form in which no differentiation between verum and placebo was possible. The study medication was linked to the patient numbers for identification according to the randomization list. Participants and care providers were blinded to the treatment.

### Statistical methods

Data are presented as mean ± standard deviation, median (25^th^ to 75^th^ percentile) or count and relative frequency. Differences between the study groups were assessed using the Fisher’s exact or the Student’s *t*-test, as appropriate. We performed a number of sensitivity analyses using different metrics for the CIPNM, including the difference from baseline to study end, yielding virtually unchanged results (data not shown). To assess the course of CIPNM we calculated the differences of the CIPNM severity sum scores regardless of the group and compared it versus 0 in a one-sided t-test. We used the Kruskal-Wallis-Test to compare non-parametric variables between patients with signs of CIP only, CIM only and combined CIP/CIM. For data management and calculations we used Excel 2011 and Stata 11.0 for Mac (Stata Corporation, College Station, TX, USA). A two-sided *P*-value ≤0.05 was generally considered statistically significant.

### Ethical approval

The study was approved by the Ethics Committee of the Medical University of Vienna. According to Austrian law and the guidelines of the research ethics committee, written informed consent was obtained from patients after they regained consciousness.

## Results

**Figure 2:**
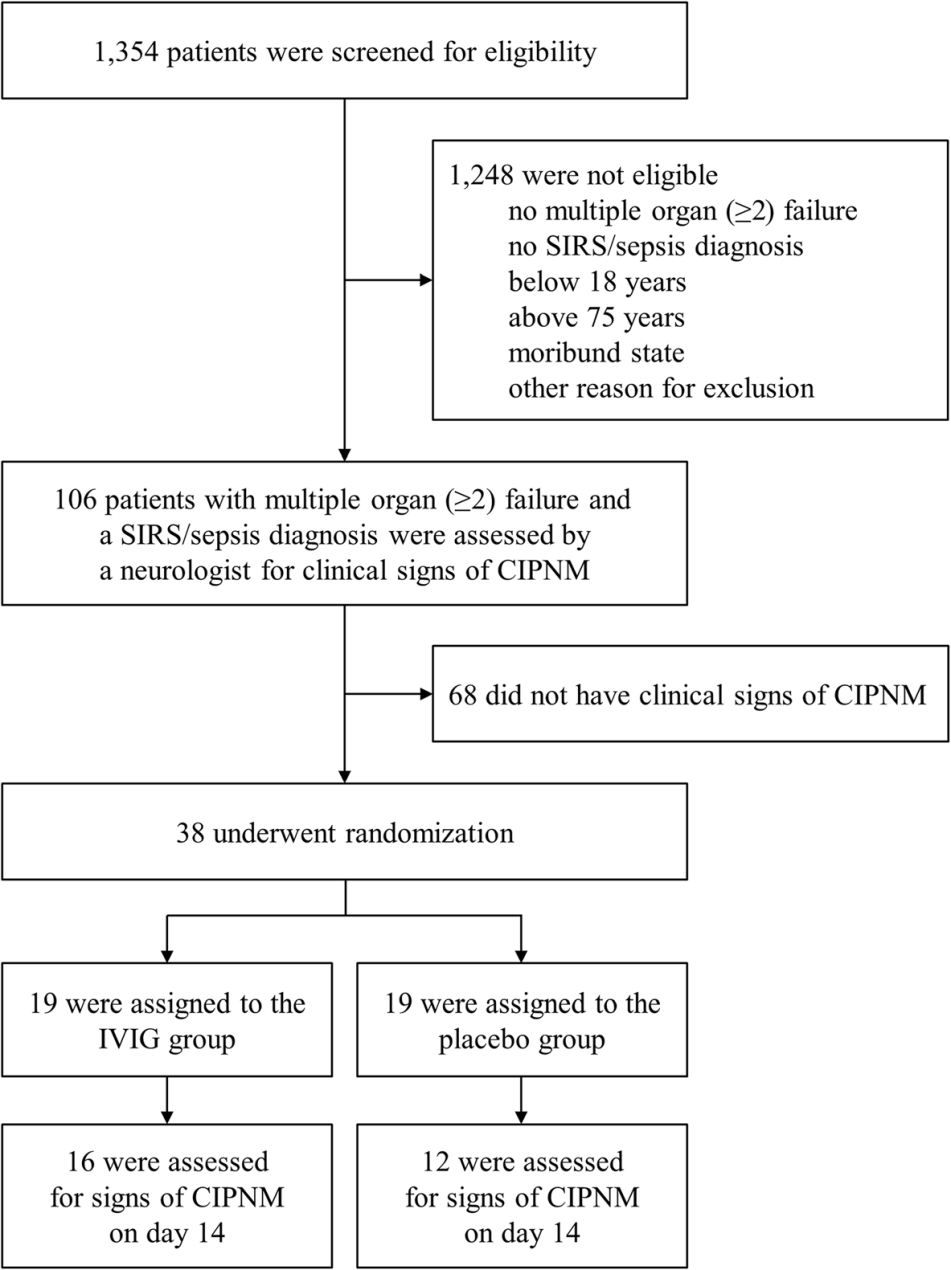
**Screening and randomization scheme.** Patients were screened for multiple (≥2) organ failure and a SIRS/sepsis diagnosis. Patients meeting these criteria were assessed by a neurologist for clinical signs of critical illness polyneuropathy and/or myopathy (CIPNM). Patients with clinical signs of CIPNM were randomized to receive either intravenous immunoglobulins (IVIG) or placebo.

A total of 38 critically ill patients with MOF, a SIRS/sepsis diagnosis and clinical signs of CIPNM were recruited between December 2004 and April 2009 and randomized to either receive IVIG or placebo (Figure 2). The study team determined during the first interim analysis that the trial be terminated due to futility in reaching the primary endpoint. This decision was based on similar CIPNM scores in the intervention and control group after enrollment of 38 patients. Nineteen patients were treated with IVIG and 19 with placebo for three consecutive days, respectively. Treatment was started at a median five (three to seven) days after the onset of SIRS/sepsis. There were no significant differences in baseline characteristics between the two study groups. CIP, CIM and CIPNM scores in both study groups were markedly increased on baseline confirming the signs of CIPNM found in the clinical examination (Table 1).

**Table 1:** Admission reason and patients’ characteristics

	**IVIG (n = 19)**	**Placebo (n = 19)**	***P*-value**
**Admission reason**	**Number of patients**		
Respiratory failure	7	8	1.00
Cardiopulmonary resuscitation	1	2	1.00
Sepsis/septic shock	9	8	1.00
Cardiogenic shock	1	1	1.00
Coma	1	0	1.00
Age (years)	61 ± 11	66 ± 12	0.18
Gender (female/male)	(7/12)	(10/9)	0.52
BMI (kg/m^2^)	28 ± 5	28 ± 6	0.94
SOFA score	11 ± 4	11 ± 5	0.88
APACHE III score	96 ± 28	96 ± 24	0.99
Mortality on Day 14 (non-survivors (%))	3 (16%)	3 (16%)	1.00
Mortality on Day 28 (non-survivors (%))	5 (26%)	6 (32%)	1.00
Length of ICU stay (days)	30 ± 16	27 ± 13	0.66
CIP score on Day 0	2.6 ± 1.5	2.0 ± 1.3	0.26
CIM score on Day 0	1.1 ± 0.8	1.4 ± 0.8	0.27
CIPNM severity sum score on Day 0	3.6 ± 1.8	3.4 ± 1.5	0.71
C-reactive protein (mg/dL) on Day 0	13 ± 11	13 ± 8	0.99
Fibrinogen (mg/dL) on Day 0	462 ± 186	563 ± 192	0.11
Leukocytes (G/L) on Day 0	19.4 ± 10.3	18.6 ± 8.7	0.78

Data are means ± SD or absolute counts. APACHE III, Acute Physiology and Chronic Health Evaluation III; BMI, body mass index; CIM, Critical illness myopathy; CIP, Critical illness polyneuropathy; CIPNM, Critical illness polyneuropathy and/or myopathy; IVIG, intravenous immunoglobulin; SOFA, Sequential Organ Failure Assessment score.

**Figure 3:**
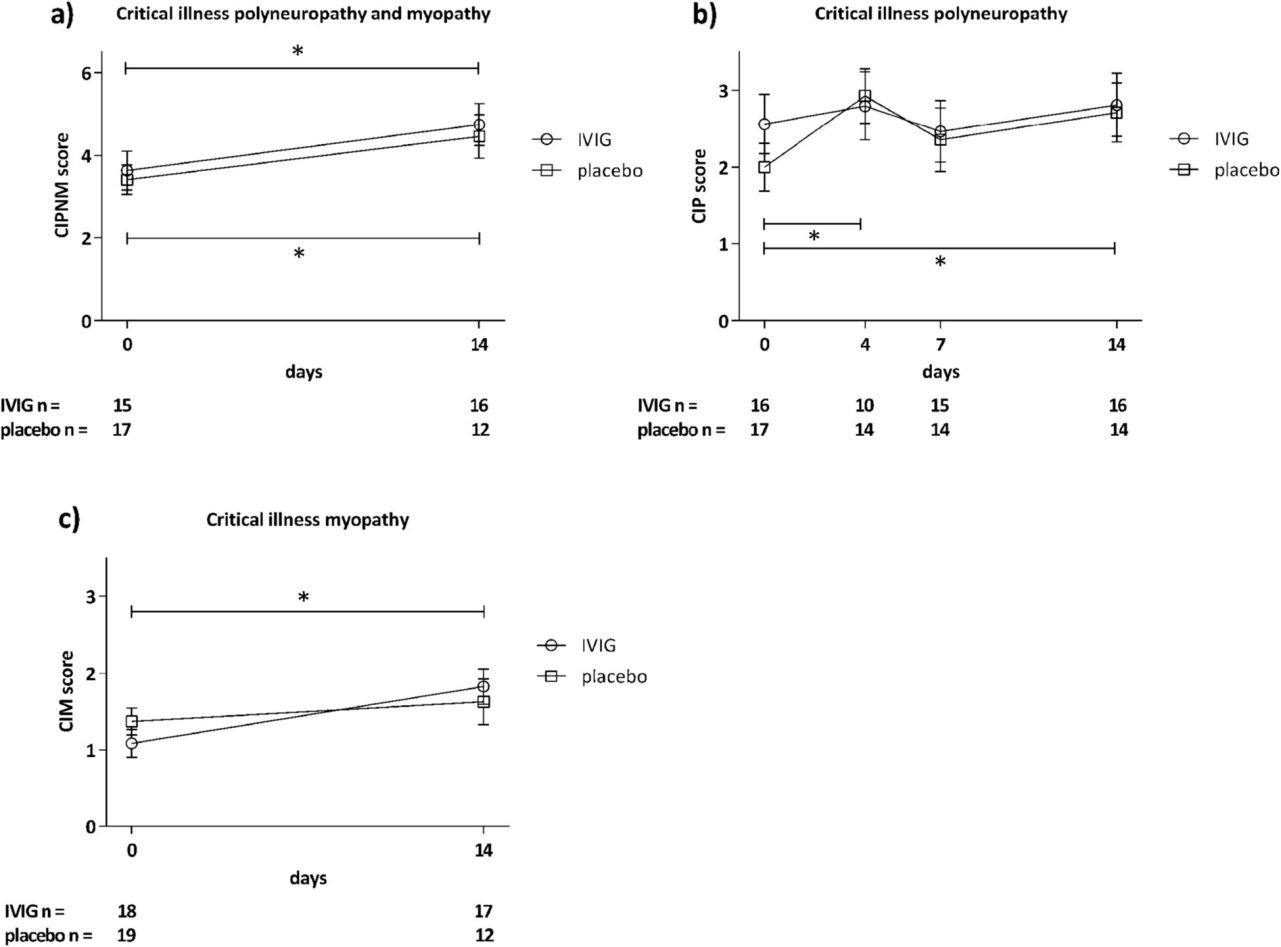
**Critical illness polyneuropathy, myopathy and critical illness polyneuropathy and/or myopathy scores.** Critical illness polyneuropathy and/or myopathy (CIPNM) severity sum score was not different on Day 14 between the intravenous immunoglobulin (IVIG) and placebo treatment group based on electrophysiological stimulation and muscle histology. CIPNM severity sum score deteriorated from baseline (Day 0) to Day 14 in both groups **(a)**. Critical illness polyneuropathy **(**CIP) was similar in the IVIG and placebo treatment group at all times based on electrophysiological stimulation **(b)**. Critical illness myopathy (CIM) scores were similar in the two groups based on muscle histology **(c)**. A two-sided *P*-value ≤0.05 was considered statistically significant (*).

The primary outcome CIPNM severity sum score on Day 14, as assessed by a combination of EPS of the ulnar, median and tibial nerves and histological examination of a muscle biopsy were not statistically different between the two groups (Figure 3a).

Moreover, neither isolated findings of EPS on days 0, 4, 7 and 14 (CIP score) nor of the histological examination of muscle biopsies on days 0 and 14 (CIM score) differed between the two groups on any of the time points (Figures 3a, b). Similarly, the secondary outcomes 28-day mortality and length of ICU stay were similar between the groups (Table 1). CIPNM deteriorated significantly from Day 0 to Day 14 regardless of the group allocation (Figure 3a).

At baseline, 16% of the patients (5/32) presented with increased CIP scores only, 16% (5/32) with increased CIM scores only, and 66% (21/32) with a combination of increased CIM and CIP scores. Thus, 97% of the analyzed patients (31/32) were diagnosed with CIPNM at baseline based on EPS and muscle histology findings.

In six patients, either the CIM or CIP score was not available, and one patient did not show signs of CIM or of CIP at baseline. Age, length of ICU stay, Sequential Organ Failure Assessment score (SOFA) score, APACHE III score and mortality were similar between patients having CIM, CIP or combined CIP/CIM.

EPS and histological assessment could not be performed at some time points due to the following reasons: patient’s death (histology on Day 14 (6 times) and EPS on days 4, 7, 14 (10 times)), withdrawn consent (histology on day 14 (once)), prone position (EPS on days 4, 7, 14 (3 times)), insufficient muscle tissue in biopsy (histoogy on days 0 and 14 (3 times)), necrotizing fasciitis (EPS on days 0, 4 (twice)), dislocated fracture (EPS on days 4, 7, 14 (3 times)), or logistic reasons (EPS on days 4, 7 (18 times)).

## Discussion

CIPNM is a serious complication of critically ill patients leading to muscle weakness and weaning failure. To date, no specific treatment has been proven in randomized controlled trials to prevent or mitigate CIPNM [1]. As there is evidence for a role of immune mechanisms in CIPNM [3], Wijdicks *et al.* administered IVIG in three patients, without beneficial effects [20]. However, in a retrospective analysis of 33 patients early administration of IgM-enriched IVIG was suggested to prevent CIPNM [15].

The present study is the first prospective, randomized, double-blinded, placebo-controlled trial assessing the impact of IgM-enriched IVIG on CIPNM. To achieve a potentially optimal effect of IVIG, we included only severely ill patients with MOF, a SIRS/sepsis diagnosis and clinical signs of CIPNM.

However, IVIG did not mitigate CIPNM in the critically ill patients in the present trial. Neither CIP as determined by EPS of three nerves on days 0, 4, 7 and 14 nor CIM as assessed by the histological examination of muscle biopsies on days 0 and 14 were different in the IVIG group compared to the controls at any time point. Moreover, length of ICU stay and mortality were similar in both groups.

More than two-thirds of the patients presented with both increased CIM and CIP scores while 16% had either elevated CIP or CIM scores. Thus, 97% of the patients (31/32) presented with CIPNM at baseline based on EPS and muscle histology findings. This is comparable to patients with severe sepsis [10]. As CIP and CIM are overlapping diseases, the CIP (CIM) score does not necessarily reflect severity of CIP (CIM) only, but should be seen as a marker of the severity of CIPNM.

Mohr *et al.* found some evidence in a retrospective chart analysis of IVIG being able to prevent CIPNM in critically ill patients using EPS [15]. Based on the retrospective character of their analysis, the evidence has been regarded as weak and is in contrast with findings of our prospective, randomized, double-blinded placebo-controlled trial. However, Mohr *et al.* started their IVIG treatment within 24 hours after onset of sepsis/multi organ failure and did not wait for the first clinical signs of CIPNM. As we administered IVIG only after the first clinical evidence of CIPNM at median five (three to seven) days after the start of the respective SIRS/sepsis episode, these two studies are not entirely comparable.

### Rationale for the treatment strategy

The pathophysiologic rationale for using IVIG to treat CIPNM in the present study is based on the association of CIPNM with pro-inflammatory cytokines, such as TNF-α, IFN-γ, IL-1, and IL-12 accompanied by increased E-selection expression [3,17]. This is suggested to promote the adhesion of leukocytes to endothelial-cells and extravasation of activated leukocytes within the endoneurial space. The increased cytokine production leads to enhanced vascular permeability favoring the passage of neurotoxic factors into the endoneurium causing neuron damage [18]. Furthermore, elevated cytokine levels directly induce muscle protein damage via activation of calpain and ubiquitine-proteasome [14]. The anti-inflammatory and immunomodulating properties of IVIG are mediated by regulating the production, release and function of pro-inflammatory cytokines and have been successfully used in numerous autoimmune and inflammatory diseases [16,21,22].

The use of IgM-enriched IVIG was based on the potential superiority over standard IVIG as seen in sepsis treatment and on the analysis of Mohr *et al.,* who suggested a beneficial effect of IgM-enriched IVIG in the prevention of CIPNM [15,23].

Standard IVIG has been safely administered intravenously at daily doses of 0.40 g/kg body weight over five days in patients with Guillain-Barré syndrome [24]. Mohr *et al.* administered IgM-enriched IVIG at doses of 0.3 g/kg body weight daily over three days [15]. However, the manufacturer recommends that IgM-enriched IVIG be administered at a maximum dose of 0.25 g/kg body weight daily for three consecutive days, which is also the common dosage for the treatment of severe sepsis [25]. Therefore, we decided to administer IgM-enriched IVIG at a dose of 0.25 g/kg body weight daily for three consecutive days. Nevertheless, we cannot rule out a potential benefit with higher doses of IgM-enriched IVIG regarding the treatment of CIPNM.

### Strengths and limitations

It is desirable to have a clinical endpoint like the Medical Research Council (MRC) scale for muscle strength to assess the course of CIPNM. However, the MRC scale assessment depends on patient's cooperation and cannot be performed in patients who are not fully awake [26]. Patients in our study were severely ill, represented by MOF, SIRS/sepsis and high SOFA/APACHE III scores. The vast majority was fully or partly sedated (87% at baseline; 50% on Day 14) and/or intubated/tracheotomized (95% at baseline; 84% on Day 14). Therefore, clinical assessment of the muscle weakness using the MRC scale was not feasible in the majority of our patients. At baseline only 5 of 38 patients (3 in the placebo, 2 in the IVIG group) were cooperative enough to allow the clinical assessment of muscle strengths using the MRC scale.

This is similar to Routsi *et al.,* who only could determine the MRC score in one-third of their patients although their patients were less ill than those in the present study [26]. Thus, as a clinical endpoint was not feasible, we regarded the CIPNM severity sum score, based on serial EPS and two muscle biopsies, as the most appropriate method of assessing the course of CIPNM in critically ill patients who are not fully awake. A total of 106 patients fulfilling the screening criteria (SIRS/Sepsis and MOF) were evaluated by a neurologist in order to only randomize patients with clinical signs of CIPNM. This evaluation was challenging as the majority of the patients was not fully awake. However, unlike the MRC scale assessment, its aim was not to measure CIPNM using a metric scale but to select patients with an advanced stage of CIPNM. One-third (38 of 106) of patients met these criteria. CIPNM was confirmed in 97% (37 of 38) of patients at baseline based on EPS and muscle histology findings with relatively high CIPNM sum scores. Therefore, we regard the initial clinical evaluation as a valid tool to specifically select patients with an advanced stage of CIPNM, whereas the sensitivity of this evaluation may have been rather low [1].

The differentiation between CIP and CIM is often not possible in critically ill patients by EPS alone. This shortcoming also could be compensated for by using the CIPNM severity sum score. Routsi *et al.,* who suggested that electrical muscle stimulation may prevent CIPNM, used only a clinical score for muscle strength to assess CIPNM and, therefore, could not differentiate between CIP and CIM [26]. Another method to make a distinction between CIP and CIM is direct muscle stimulation [27]. However, as muscle biopsy is regarded as the gold standard, we did not use direct muscle stimulation in our study [4].

Van den Berghe *et al.* found a reduced incidence of CIPNM in a pre-planned subgroup analysis of critically ill patients treated with IIT compared to conventional insulin therapy. Similarly, no differentiation between CIP and CIM was feasible in their study, as no histological assessment was done [13].

It has been controversially discussed if discrimination between CIP and CIM is reasonable. However, exact differential diagnosis between these two entities leads to better prognostic information regarding long term disability [1,28]. CIM in combination with CIP is associated with a more severe weakness and longer ICU length of stay than CIM alone [29]. Moreover, CIM has a better long-term prognosis than CIP [30].

The main limitation of the present trial is the relatively small number of critically ill patients included in our trial prone to type II errors. Although we did not see any differences in the outcomes between the groups, this cannot entirely rule out a (small) effect of IVIG on CIPNM.

Furthermore, not all EPS or muscle biopsy evaluations could be performed as scheduled. The recruitment period of 4.5 years is rather long for a single-center trial and this potentially influenced the results. The slow recruiting is attributed to the very specific inclusion criteria, based on which only patients with a two-or-more organ failure, SIRS/sepsis, and clinical evidence for CIPNM could be included. To minimize the potential bias of the relatively long recruitment period we ensured that all procedures were carried out by the same team throughout the study period.

Although EPS and muscle biopsy are the methods of choice of assessing nerve and muscle damage in CIPNM [4], a combination of both (CIPNM sum score) as used in the present study has never been validated to be superior. Therefore, we also provide separate results of EPS and muscle biopsy assessment which do not differ from the CIPNM sum score (Figure 3). The CIPNM sum score should be further validated in future trials for determining the specificity and sensitivity of CIPNM in critically ill patients.

Another limitation may have been the use of the “CIM score” based on the histological assessment of muscle biopsies. Although histological assessment is the diagnostic method of choice to evaluate myopathy in critically ill patients the grading of the “CIM score” is only semi-quantitative and has not been validated before. Muscle biopsy is regarded as safe and well tolerated in critically ill patients but it is still an invasive procedure [31]. Therefore, we suggest that muscle biopsies should primarily be used in clinical trials. Unclear muscle weakness and inconclusive electrophysiological findings may justify muscle biopsy in the clinical routine.

Ultrasound has been successfully used to reliably measure muscle mass in critically ill patients [32]. However, at the start of enrollment (December 2004), this information was not yet available. Furthermore, the patients included in our trial were more severely ill than in the trial of Gruther *et al.* As tissue edema is common in severely ill patients, the assessment of the muscle mass using ultrasound may be challenging. Nevertheless, ultrasound examination should be considered as additional outcome in future trials.

Patients with clinical unapparent polyneuropathy or mild polyneuropathy were eligible for enrollment, as we did not expect an effect on the primary outcome.

We hypothesize that the following circumstances may be responsible for the lack of effect of IVIG. First, we decided to include patients that were already presenting with clinical signs of CIPNM at an early stage to achieve a maximal effect of IVIG. However, the application of IVIG at an even earlier time point - when the first signs of CIPNM can be verified only using electrophysiology measures may result in improved effects of IVIG. This was similarly observed in patients with severe sepsis, who had a significantly improved survival rate when IVIGs were administered early compared to at a more advanced phase of sepsis [33].

Thus, earlier or even prophylactic application of IVIG may show better effects of IVIG regarding the prevention or mitigation of CIPNM, since a short, albeit crucial, time period may pass between first nerve and/or muscle fiber damage and first demonstrable electrophysiological changes, not to mention the first clinical signs, chosen as inclusion criterion in our study. However, a prophylactic treatment had required the inclusion of a lot more patients.

Potentially, a beneficial effect of IVIG on CIPNM may only be seen months after ICU discharge and was still concealed on Day 14 when we assessed the primary outcome. Due to patients lost to follow-up, this requires the inclusion of a higher number of patients.

Furthermore, the pathophysiology of CIPNM is complex and a multimodal cause is postulated. This includes alterations of the local immunity, decreased microcirculation of peripheral nerves, increased generation and deficient scavenging of reactive oxygen species, enhanced permeability for neurotoxic factors into the endoneurium, direct muscular protein breakdown and acquired channelopathy [34]. However, IVIG has only a relatively limited point of action by modulating the local immunity [16]. Thus, a multimodal therapy approach may be necessary to improve CIPNM.

## Conclusions

This prospective, randomized, double-blinded, placebo-controlled trial showed that early treatment with IVIG does neither significantly improve CIPNM nor influence length of ICU stay or mortality in critically ill patients. CIPNM deteriorated during the course of disease in critically ill patients with MOF and a diagnosis of SIRS/sepsis.

## Key message

• Early treatment with IVIG does not improve CIPNM in critically ill patients with MOF and SIRS/sepsis.

## Abbreviations

APACHE III: Acute physiology and chronic health evaluation III; CIM: Critical illness myopathy; CIP: Critical illness polyneuropathy; CIPNM: Critical illness polyneuropathy and/or myopathy; CMAP: Compound muscle action potential; EMG: Electromyography; EPS: Electrophysiological stimulation; ICU: Intensive care unit; IIT: Intensive insulin therapy; IVIG: Intravenous immunoglobulin; MOF: Multiple organ failure; MRC: Medical Research Council; NCV: Nerve conduction velocity; SIRS: Systemic inflammatory response syndrome; SNAP: Sensory nerve action potential; SOFA: Sequential organ failure assessment score.

## Competing interests

Richard Brunner received a travel grant from Biotest Pharma GmbH, Dreieich, Germany for the ESICM congress in Lisbon, Portugal in 2012. Walter Rinner received a travel grant from Biotest Pharma GmbH, Dreieich, Germany for the ISICEM congress in Brussels, Belgium in 2010. For the remaining authors no conflicts of interest were declared.

## Authors’ contributions

RB collected data, carried out the statistical analyses and interpretations, and drafted the manuscript. WR performed the neurologic examinations, EPS testing and obtained muscle biopsies. RK and JW screened/enrolled patients and made substantial contributions to acquisition of data. CH evaluated the histology of muscle biopsies. HH carried out statistical analyses. CM, UH and TS made substantial contributions to the conception and design of the study. CM and UH helped to draft the manuscript. All authors read and approved the final manuscript.
